# Supplementary material for: The Correlated Beta Dose Optimisation Approach: Optimal Vaccine Dosing Using Mathematical Modelling and Adaptive Trial Design
Source: Vaccines (Basel). 2022 Oct 30;10(11):1838. doi: 10.3390/vaccines10111838 (PMC9693615; doi:10.3390/vaccines10111838)
Supplement: Supplementary file 1 [file vaccines-10-01838-s001.zip › vaccines-1907957-supplementary.pdf]

Supplementary material for paper 5

S1 EffTox Utility Function

The 'utility contour' utility function was used in the main body of the work to represent an example utility function that could be used to quantitatively define the trade-off between efficacy and toxicity. This utility function was first described by Thall and Cook in 2004[1]. This was further described by Brock in 2017 [2].

$$U(p_{eff}, p_{tox}) = 1 - ((\frac{1 - p_{eff}}{1 - anchor_{eff}})^{rho} - (\frac{1 - p_{tox}}{1 - anchor_{tox}})^{rho})^{\frac{1}{rho}}$$

With parameters $anchor_{eff}, anchor_{tox}, rho$. Clinicians are queried for the smallest value for the vaccine's efficacy probability such that, if the probability of toxicity for that vaccine was zero, the vaccine would see clinical use. This efficacy probability value is $anchor_{eff}$. Clinicians are queried for the maximum value for the vaccine's toxicity probability such that, if the probability of efficacy for that vaccine was one, the vaccine would see clinical use. This toxicity probability value is $anchor_{tox}$. Clinicians are then asked to suggest an efficacy probability and a toxicity probability p^*_{eff}, p^*_{tox} with $anchor_{eff} < p^*_{eff} < 1$ and $0 < p^*_{tox} anchor_{tox}$, where a vaccine with these efficacy and toxicity probabilities would also only be on the threshold of clinical viability.

These questions define three points in the efficacy-toxicity outcome space, $(anchor_{eff}, 0), (1, anchor_{tox}), (p^*_{eff}, p^*_{tox})$

These three points then can be used to define the neutral-utility curve. That is to say, $U(anchor_{eff}, 0) = U(1, anchor_{tox}) = U(p^*_{eff}, p^*_{tox}) = 0$.
$U(anchor_{eff}, 0) = U(1, anchor_{tox}) = 0$ is guaranteed for all values of rho, so we solve numerically

$$U(p^*_{eff}, p^*_{tox}) = 1 - ((\frac{1 - p^*_{eff}}{1 - anchor_{eff}})^{rho} - (\frac{p^*_{tox}}{anchor_{tox}})^{rho})^{\frac{1}{rho}} = 0$$

to calculate rho.

In this work we used $anchor_{eff}, anchor_{tox}, rho$ parameters from [2]. These were $anchor_{eff} = 0.4, anchor_{tox} = 0.7, rho = 2.07$, based on the points $(0.4, 0), (1, 0.7), (0.5, 0.4)$. Whilst these were not elicited from clinicians in a vaccine context, we choose to use these as they had been previously used in a clinical setting. Figure S1 shows the utility contour that arises from these parameters.

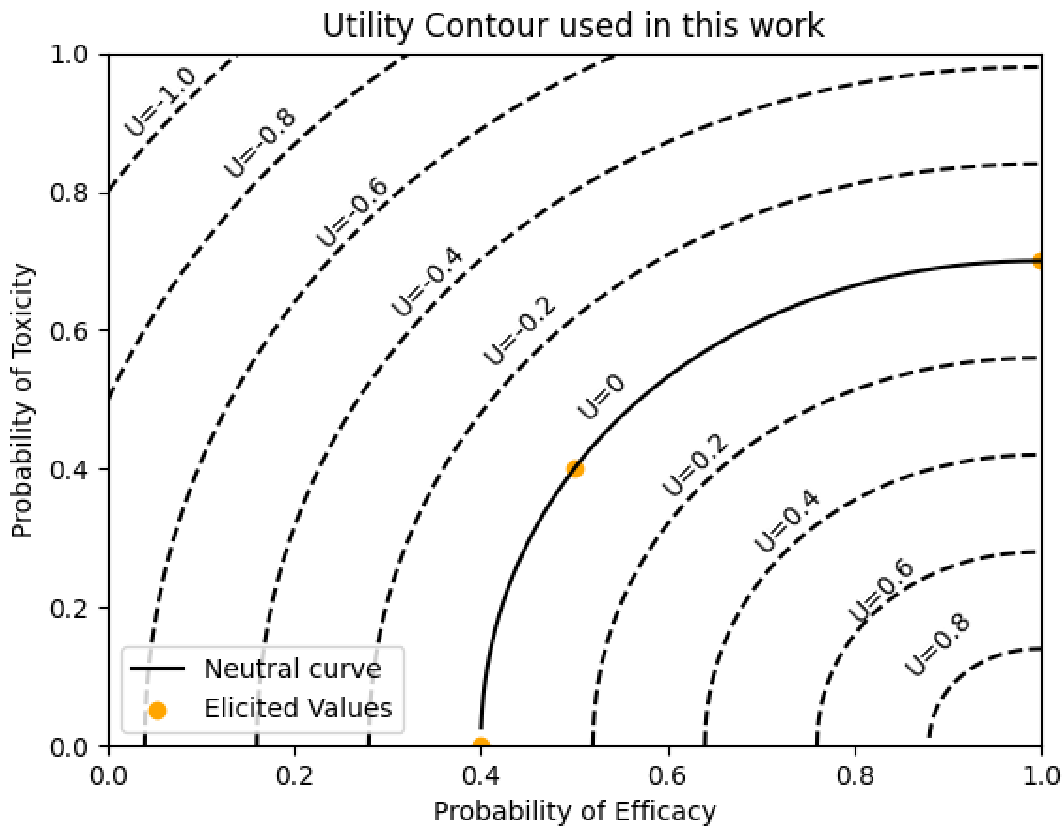

Figure S1. Utility contour used in this work. The thin black line shows the neutral utility curve. The orange dots show the three points used to define the neutral utility curve and function; (0.4, 0),(1,0.7),(0.5, 0.4).

S2 Length hyperparameters

In values were chosen using a geometric argument and validated empirically. See S7.1.2. For details.

S3 Pseudo-data

Pseudo-data or ‘anchor points’ were used to stabilise and inform models for which little real data is available. In short, we pretended in all Parametric DOAs that there

existed data which did not actually exist, but also down weighted these data during model calibration such that these data points were less important than real data.

S3.1. Efficacy Models

For single administration efficacy modelling, pseudo-data were of the form in table S.3.1.1. Thus, there were 30 pseudo individuals divided evenly over 3 doses. The weight of these datapoints was 0.05. Thus, the effective sample size of the pseudodata was 1.5 (=30x0.05), which was quickly minimal relative to the amount of real data. For example, after the first cohort the model would only be 20% influenced by these pseudo-data (1.5/(1.5+6) = 0.2) and hence 80% influenced by the ‘real’ (simulated) data.

Dose	Non-efficacy response	Efficacy Response
0.1	9	1
0.5	5	5
0.9	1	9

Table S.3.1.1. Efficacy pseudodata for single administration

For prime-boost administration efficacy modelling, pseudo-data were of the form in table S.3.1.2. Thus, there were 50 pseudo individuals divided evenly over 5 doses. The weight of these datapoints was 0.03. Thus the effective sample size of the pseudodata was 1.5 (=50 x 0.03), which is quickly minimal relative to the amount of real data.

Dose	Non-efficacy response	Efficacy Response
0,0	9	1
0,1	5	5
1,0	5	5
1,1	1	9
0.5, 0.5	5	5

Table S.3.1.2. Efficacy pseudodata for prime/boost administration

For prime-boost administration efficacy modelling, pseudo-data were of the form in table S.3.1.3. Thus there were 80 pseudo individuals divided evenly over 8 doses. The weight of these data points was 0.01875. Thus the effective sample size of the pseudodata was 1.5 (=80x0.01875), which is quickly minimal relative to the amount of real data.

Dose	Non-efficacy response	Efficacy Response
0,0,0	1	9
0,0,1	5	5
0,1,0	5	5
0,1,1	5	5
1,0,0	5	5
1,0,1	5	5
1,1,0	5	5
1,1,1	9	1

Table S.3.1.3. Efficacy pseudodata for prime/boost administration

S3.2 Toxicity Model

For single administration toxicity modelling, pseudo-data were of the form in table S.3.2.1. Thus there were 30 pseudo individuals divided evenly over 3 doses. The weight of these datapoints was 0.05. Thus the effective sample size of the pseudodata was 1.5 (=30x0.05), which was quickly minimal relative to the amount of real data.

Dose	Non-toxic response	Toxic Response
0.1	9	1
0.5	5	5
0.9	1	9

Table S.3.2.1.Toxicity pseudodata for single administration

For prime-boost administration toxicity modelling, pseudo-data were of the form in table S.3.2.2. Thus, there were 50 pseudo individuals divided evenly over 5 doses. The weight of these datapoints was 0.03. Thus the effective sample size of the pseudodata was 1.5 (=50x0.03), which was quickly minimal relative to the amount of real data.

Dose	Non-efficacy response	Efficacy Response
0,0	9	1
0,1	5	5
1,0	5	5
1,1	1	9
0.5, 0.5	5	5

Table S.3.2.2.Toxicity pseudodata for prime/boost administration

S4 SoftMax Selection Method

In this section of the supplementary we describe the SoftMax selection method of trial dose selection. We previously discussed this in [3] but replicate this description here.

Method

SoftMax selection is a method of action selection used commonly in both multi-armed bandit problems and reinforcement learning. We provide a description of action/dose selection under this method. Let A1, A2, …., An be the n possible actions available to be taken, each with respective predicted utility U1, U2,..., Un. Then an action Ai is selected to test (dose selected to trial) with probability

$$Probability\ of\ selecting\ action\ A_i = \frac{e^{inversetemperature \times U_i}}{\sum_{j=1}^{n} e^{inversetemperature \times U_j}}$$

where inverse_temperature is a hyperparameter which controls the degree of exploration. An increased inverse temperature leads to actions with lower predicted utility having lower probability of selection. For inverse_temperature = 0, which is the

lowest possible inverse_temperature, all actions are selected with equal probability (1/n). As inverse_temperature tends to infinity, this selection method tends to only selecting the action(s) with the maximum predicted utility. A random number generator is used to select an action with these probabilities.

inverse_temperature values

For the Parametric DOA, we used inverse_temperature = 6.9. This was chosen such that a predicted difference in utility of 0.1 would have a doubled probability of selection. This is shown

$$2e^{inversetemperature \times U_i} = e^{inversetemperature \times (U_i + 0.1)}$$

$$= e^{inversetemperature \times (U_i)} e^{inversetemperature \times (0.1)}$$

$$2 = e^{inversetemperature \times (0.1)}$$

$$\ln(2) = 0.69 = 0.1 \times inversetemperature$$

$$6.9 = inversetemperature$$

This may not have been optimal values, but optimal values are likely to vary depending on the scenario. This is further discussed in S7.4.

S5 Rescaled Dosing Domains

It may be reasonable to transform the ‘raw’ value of doses before modelling. This can help with stabilising computation [4]. These rescalings or transformations are of the form

$$x_i = transform(dose_i)$$

Where x_i is the transformed dose value that will be used for modelling and again $dose_i$ is the ‘raw’ dose value. In particular, using a log10 transform is common in drugs and in vaccines and would be given by

$$x_i = transform(dose_i) = log_{10}(dose_i)$$

In this work we assume that all prime/boost/second-boost doses had been transformed using a transform such that

$$0 = transform(dose_{min})$$

$$1 = transform(dose_{max})$$

Where $dose_{min}$ and $dose_{max}$ were respectively the smallest dose that could be given for the prime/boost/second-boost doses.

Two example transforms that would have this property would be the MinMax and Log10 MinMax transforms.

Minmax:

$$x_i = transform(dose_i) = \frac{dose_i - dose_{min}}{dose_{max} - dose_{min}}$$

Log10 Minmax:

$$x_i = transform(dose_i) = \frac{log_{10}(dose_i) - log_{10}(dose_{min})}{log_{10}(dose_{max}) - log_{10}(dose_{min})}$$

If $dose_{min}$=0, then a small constant may be added to ensure that the transform is well defined. One possibility is to choose this constant to be equal to the smallest positive (>0) dose in the dosing dimension.

$$x_i = transform(dose_i) = \frac{log_{10}(dose_i + dose_{leastnonzero}) - log_{10}(dose_{min} + dose_{leastnonzero})}{log_{10}(dose_{max} + dose_{leastnonzero}) - log_{10}(dose_{min} + dose_{leastnonzero})}$$

An alternative is to choose c = 1 - $dose_{min}$ and have

$$x_i = transform(dose_i) = \frac{log_{10}(dose_i + c) - log_{10}(dose_{min} + c)}{log_{10}(dose_{max} + c) - log_{10}(dose_{min} + c)}$$

Both would distort the data, but this may be justified by improved modelling stability.

S6 Scenario Creation

Simulation studies require researchers to define scenarios that are used to evaluate the approaches that the simulation study aims to investigate. In previous simulation

studies, which evaluated dose optimisation between only a small number of dosing groups, the efficacy probabilities and toxicity probabilities for each dosing group have been chosen by hand to create scenarios with specific qualitative features (e.g. peaking dose-efficacy curves). Little detail is typically given to how these are chosen, as with only a small number of dosing groups it is easy for readers to visualise.

In this work, scenarios could have 101, 441, or 1331 potential doses that needed to have defined efficacy and/or toxicity probabilities for single, prime/boost, and prime/boost/second-boost scenarios respectively. These were too many doses to choose by hand, so we had to use an algorithmic approach to setting true efficacy/toxicity probabilities for the doses in each scenario. We considered using parametric models or kernel based algorithms to generate these probabilities, however we decided that using either parametric model or kernel based algorithms could bias our results. We choose to use a K-nearest neighbours style algorithm that involved iterative averaging, which produced smooth and continuous dose-response curves if the number of iterations was large enough,

This algorithm took the following inputs:

- The prime doses that should be used in the iterative process
- Potentially the boost doses that should be used in the iterative process
- Potentially the second-boost doses that should be used in the iterative process
- Anchor Doses: Doses that we would like to set to a specific value in the scenario
- Anchor Probabilities: The Specific probabilities for the anchor doses.
- K, the number of neighbours for each dose that will be averaged. This increases the ‘smoothness’ of the resulting curve.
- The number of iterations of the algorithm to use.

For the algorithm, all doses (potential combinations of any prime and boost doses) were found. For each of these combinations, we set an initial probability of response of 0.5. Then the following iterative process was used:

For doses which were specified as anchor doses, set the probability of response equal to the respective anchor probability for that dose.

Set the probability of response for each dose equal to the mean value of its K-nearest neighbours, which was defined by Euclidean distance and included itself.

Repeat steps 1 and 2 as many times as specified.

By conducting a large number of iterations, the probability of response for each dose would be approximately the average of its surrounding doses, and there would be smooth dose-response curves between anchor points. Where we generated probabilities for doses that were outside of the [0,1] bounded dosing domain, these doses and respective probabilities were then excluded.

Rather than giving the probabilities for each dose for each scenario in tabular format, which we don't believe would be easy to parse, for each scenario we give the input parameters used to generate the dose-response curve. We hope that this shows how we created scenarios with certain qualitative behaviours.

Objective 1, Scenario 1 Efficacy

Inputs	Values
Prime doses	[-1.00, -0.99, …, 1.99, 2.00]
Anchor doses	[-0.5], [1.5]
Anchor probabilities	[0.05], [0.95]
K	21
Iterations	11

Objective 1, Scenario 2 Efficacy

Inputs	Values
Prime doses	[0.00, 0.01, …, 0.99, 1.00]
Anchor doses	[0.0], [1.0]
Anchor probabilities	[0.05], [0.9]
K	9
Iterations	21

Objective 1, Scenario 3 Efficacy

Inputs	Values
Prime doses	[-0.50, -0.49, …, 1.49, 1.50]
Anchor doses	[-0.2], [0.1], [0.5], [0.9], [1.2]
Anchor probabilities	[0.1, 0.5, 0.8, 0.55, 0.2]
K	15
Iterations	21

Objective 1, Scenario 4 Efficacy

Inputs	Values
Prime doses	[0.00, 0.01, …, 0.99, 1.00]
Anchor doses	[0], [0.2], [0.4], [0.6], [0.7], [1]
Anchor probabilities	[0.05], [0.1], [0.15], [0.3], [0.7], [0.5]
K	5
Iterations	21

Objective 1, Scenario 5 Efficacy

Inputs	Values
Prime doses	[0.00, 0.01, …, 0.99, 1.00]
Anchor doses	[0], [0.2], [0.4], [0.6], [0.8], [1]
Anchor probabilities	[0.8], [0.7], [0.3], [0.15], [0.1], [0.05]
K	5
Iterations	21

Objective 1, Scenario 6 Efficacy

Inputs	Values
Prime doses	[0.00, 0.01, …, 0.99, 1.00]
Anchor doses	[0.0], [0.2], [0.55], [0.65], [1.0]
Anchor probabilities	[0.3], [0.5], [0.7], [0.5], [0.7]
K	5
Iterations	21

Objective 1, Scenario 7 Efficacy

Inputs	Values
Prime doses	[0.00, 0.01, …, 0.99, 1.00]
Anchor doses	[0.0], [0.5], [1.0]
Anchor probabilities	[0.75], [0.85], [0.75]
K	11
Iterations	11

Objective 2, Scenario 1 Efficacy

Inputs	Values
Prime doses	[0.00, 0.05, …, 0.95, 1.00]
Boost doses	[0.00, 0.05, …, 0.95, 1.00]
Anchor doses	[1,1], [0,0], [0,1], [1,0], [0.6, 0.6]
Anchor probabilities	[0.5], [0.1], [0.4], [0.4], [0.9]
K	15
Iterations	9

Objective 2, Scenario 2 Efficacy

Inputs	Values
Prime doses	[0.00, 0.05, …, 0.95, 1.00]
Boost doses	[0.00, 0.05, …, 0.95, 1.00]
Anchor doses	[1,1], [0,0], [0,1], [1,0]
Anchor probabilities	[0.5], [0.1], [0.8], [0.9]
K	9
Iterations	11

Objective 2, Scenario 3 Efficacy

Inputs	Values
Prime doses	[0.00, 0.05, …, 0.95, 1.00]
Boost doses	[0.00, 0.05, …, 0.95, 1.00]
Anchor doses	[1,1], [0,0], [0,1], [1,0]
Anchor probabilities	[0.95], [0.05], [0.6], [0.6]
K	9
Iterations	11

Objective 2, Scenario 4 Efficacy

Inputs	Values
Prime doses	[0.00, 0.05, …, 0.95, 1.00]
Boost doses	[0.00, 0.05, …, 0.95, 1.00]
Anchor doses	[1,1], [0.9,.9],[0,0], [0,1], [1,0]
Anchor probabilities	[0.6], [0.95], [0.05], [0.4], [0.6]
K	9
Iterations	11

Objective 2, Scenario 5 Efficacy

Inputs	Values
Prime doses	[0.00, 0.05, …, 0.95, 1.00]
Boost doses	[0.00, 0.05, …, 0.95, 1.00]
Anchor doses	[1,1], [1,0.5], [1,0], [0.9,1], [0.9,0.5], [0.9,0], [0,1], [0,0.5], [0,0]
Anchor probabilities	[0.8], [0.7], [0.7], [0.9], [0.8], [0.7], [0.1], [0.05], [0.05]
K	9
Iterations	10

Objective 2, Scenario 6 Efficacy

Inputs	Values
Prime doses	[0.00, 0.10, …, 0.90, 1.00]
Boost doses	[0.00, 0.10, …, 0.90, 1.00]
Second-boost doses	[0.00, 0.10, …, 0.90, 1.00]

Anchor doses	[0,0,0], [0,0,1], [0,1,0], [0,1,1], [1,0,0], [1,0,1], [1,1,0], [1,1,1], [0.7, 0.1, 0.3], [0.7, 0.1, 0.3], [0.7, 0.1, 0.3]
Anchor probabilities	[0], [0], [0], [0], [0], [0], [0], [0], [0.9], [0.9], [0.9]
K	7
Iterations	5

Objective 2, Scenario 7 Efficacy

Inputs	Values
Prime doses	[0.00, 0.10, …, 0.90, 1.00]
Boost doses	[0.00, 0.10, …, 0.90, 1.00]
Second-boost doses	[0.00, 0.10, …, 0.90, 1.00]
Anchor doses	[0,0,0], [0,0,1], [0,1,0],<br>[0,1,1] , [1,0,0], [1,0,1],<br>[1,1,0], [1,1,1], [0.5,0,0]
Anchor probabilities	[0.1], [0.5], [0.5],<br>[0.8], [0.1], [0.9],<br>[0.9], [0.4], [0.1]
K	27
Iterations	3

Objective 3, Scenario 1 Efficacy

As objective 1 scenario1.

Objective 3, Scenario 1 Toxicity

Inputs	Values
Prime doses	[-1.00, -0.99, …, 1.99, 2.00]
Anchor doses	[0.2], [1.5]
Anchor probabilities	[0.05], [0.65]
K	21
Iterations	11

Objective 3, Scenario 2 Efficacy

As objective 1 scenario 4.

Objective 3, Scenario 2 Toxicity

Inputs	Values
Prime doses	[-1.00, -0.99, …, 1.99, 2.00]
Anchor doses	[0.7], [0.8]
Anchor probabilities	[0.05], [0.95]
K	3
Iterations	11

Objective 3, Scenario 3 Efficacy

As objective 1 scenario 1.

Objective 3, Scenario 3 Toxicity

As objective 3 scenario 1.

Objective 3, Scenario 4 Efficacy

As objective 1 scenario 4.

Objective 3, Scenario 4 Toxicity

As objective 3 scenario 2.

Objective 3, Scenario 5 Efficacy

As objective 2 scenario 3.

Objective 3, Scenario 5 Toxicity

Inputs	Values
Prime doses	[0.00, 0.05, …, 0.95, 1.00]
Boost doses	[0.00, 0.05, …, 0.95, 1.00]
Anchor doses	[1,1], [0,0], [0,1], [1,0], [0.5, 0.5], [0, 0.5], [0.5,0], [1, 0.5], [0.5,1]
Anchor probabilities	[0.9], [0.1], [0.7], [0.8], [0.2], [0.23], [0.2], [0.85], [0.75]
K	13
Iterations	9

Objective 3, Scenario 6 Efficacy

As objective 2 scenario 2.

Objective 3, Scenario 6 Toxicity

As objective 3 scenario 5.

Objective 4

Objective 4 reused scenarios from the previous sections as detailed in the main body. Please refer to the relevant scenarios.

S7. Additional DOA Hyperparameters

S7.1. CoBe DOA: CCBP Kernel Type and Length Hyperparameters

The CoBe DOA uses a CCBP model of dose response, which relies on a kernel function to describe similarity between doses. In this work, we used the Squared Exponential kernel, as this was the kernel used by the original authors of the CCBP model. However, more recent work has suggested that other kernel functions should be considered, for example the ‘SBC’ kernel described by Rolland et al [5]. This was shown to outperform the squared exponential kernel for large amounts of data (>1000 data points). We chose to predominantly consider the squared exponential kernel due to considering typically <300 data points, but it was possible that the SBC kernel may have been superior. Additionally, both of these kernels require a length hyper parameter l to be specified. In the main body of the work, we used l = 0.2 for our squared exponential kernel, but again it is possible that this may have biased our results. We originally choose l = 0.2 as that would mean $K(d_i, d_i+0.15)=0.5$, which seemed reasonable. We did not conduct a systematic optimisation of l prior to conducting the simulation studies, as this may have biased our findings in favour of the CoBe DOA. Additionally, we believed that it would be likely that the ‘optimal’ value for l would depend on the scenario.

We did however investigate of the effect that changing the kernel function or value of the length hyperparameter may have had after the main investigation was concluded. This was done by following the same methodology and scenarios as objective 1 of the main body of this work, but where all DOAs investigated were CoBe DOAs with one of two different kernel functions, each with 4 different potential length hyperparameters. We also investigated the ‘uncorrelated’ kernel described in the main body of the text. Results are shown in Figure S2.

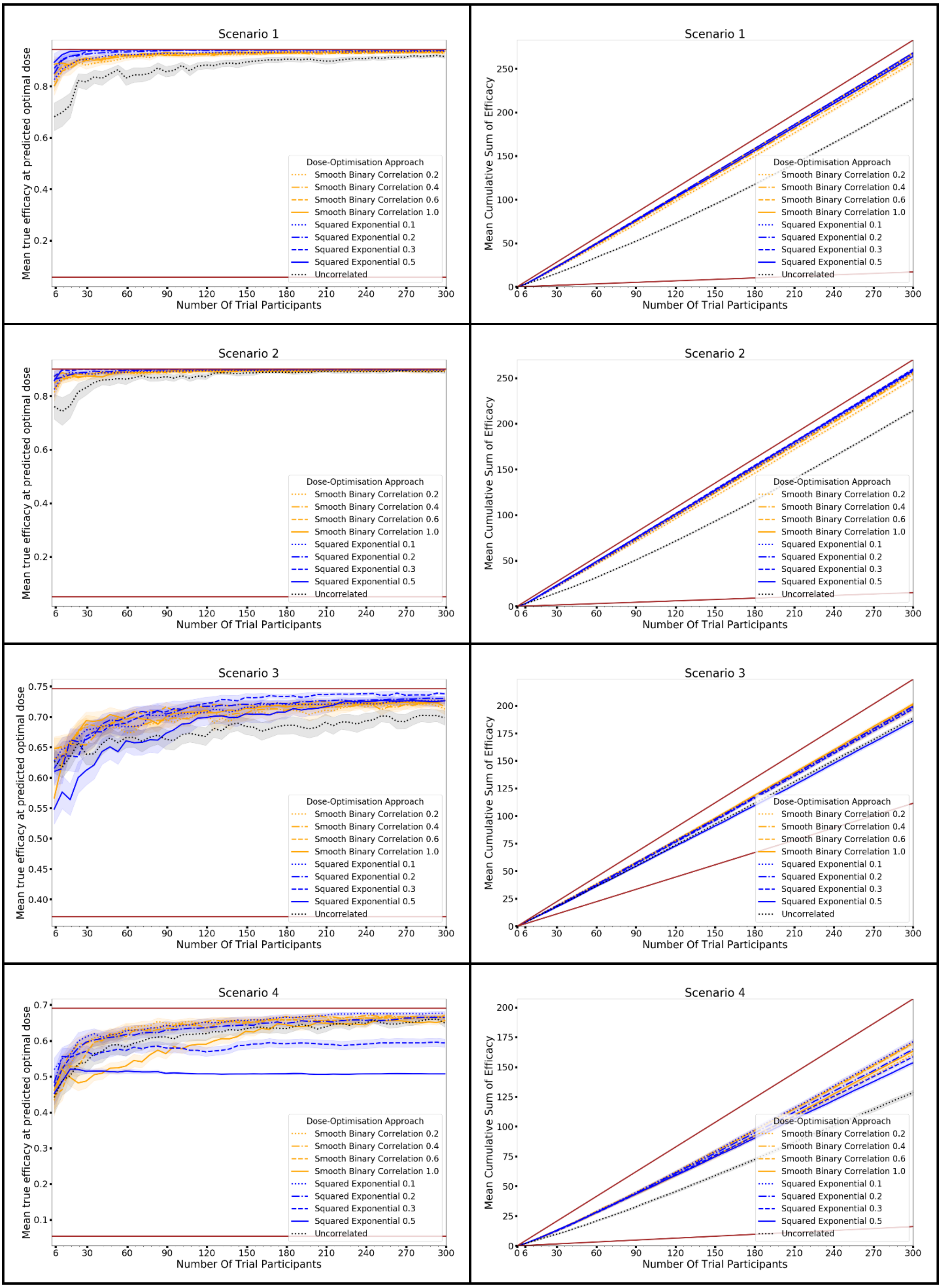

Scenario 1
Mean true efficacy at predicted optimal dose
Number Of Trial Participants
Dose-Optimisation Approach
Smooth Binary Correlation 0.2
Smooth Binary Correlation 0.4
Smooth Binary Correlation 0.6
Smooth Binary Correlation 1.0
Squared Exponential 0.1
Squared Exponential 0.2
Squared Exponential 0.3
Squared Exponential 0.5
Uncorrelated
Scenario 1
Mean Cumulative Sum of Efficacy
Number Of Trial Participants
Scenario 2
Mean true efficacy at predicted optimal dose
Number Of Trial Participants
Scenario 2
Mean Cumulative Sum of Efficacy
Number Of Trial Participants
Scenario 3
Mean true efficacy at predicted optimal dose
Number Of Trial Participants
Scenario 3
Mean Cumulative Sum of Efficacy
Number Of Trial Participants
Scenario 4
Mean true efficacy at predicted optimal dose
Number Of Trial Participants
Scenario 4
Mean Cumulative Sum of Efficacy
Number Of Trial Participants

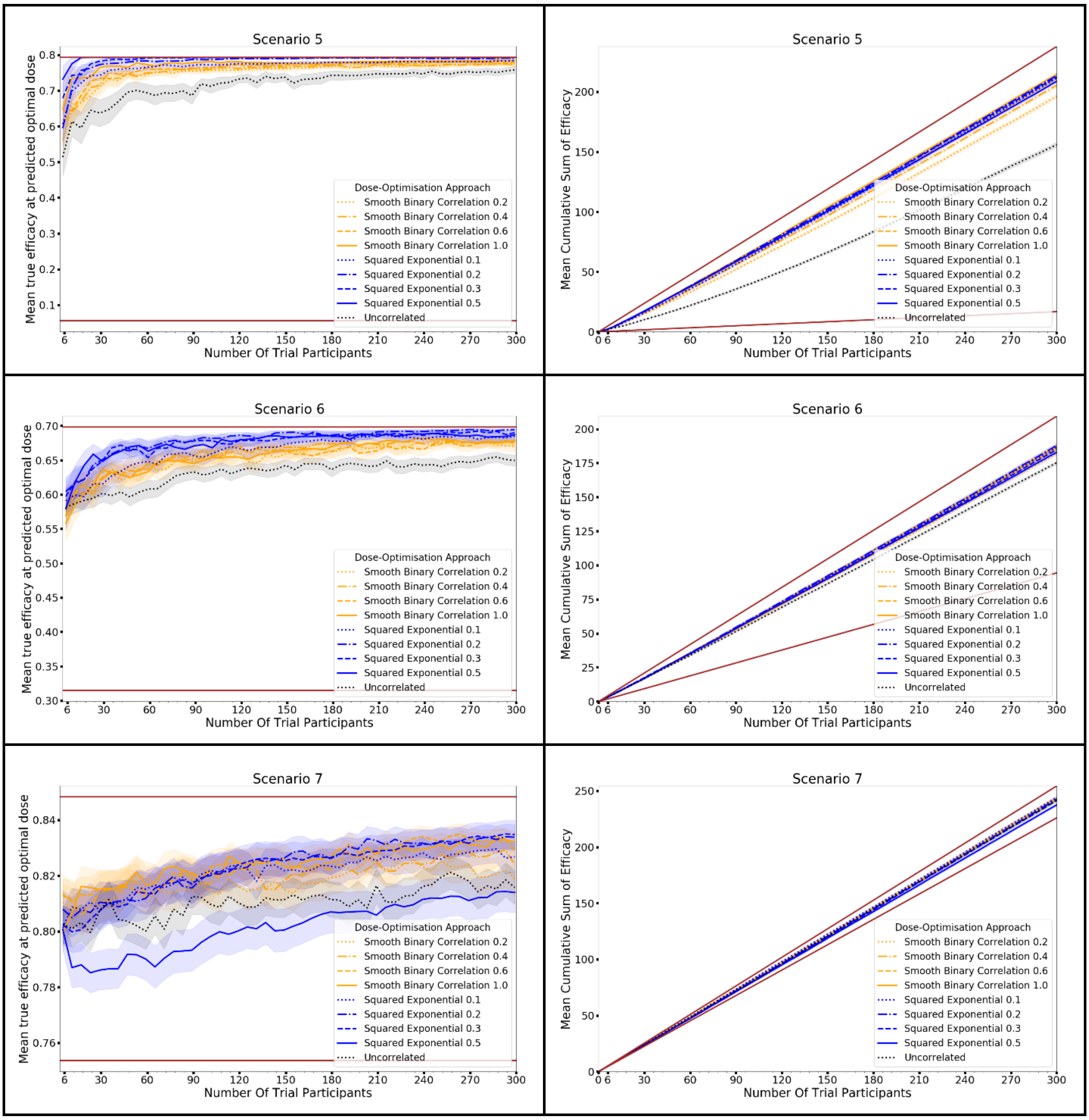

Figure S7.1. Mean true efficacy at the predicted optimal dose (left) and mean cumulative sum of efficacy (right) against trial size for all seven objective S7.1 scenarios (top to bottom). These are the mean values and 95%CI values across 100 simulations. For the true efficacy plots (left), the brown lines show the minimum and maximum possible efficacy that could be achieved in that scenario. For the cumulative efficacy plots (right) the brown lines represent the maximum and minimum cumulative efficacy sum that could be expected for that scenario.

These results suggest that using the SBC kernel rather than the squared exponential kernel would not have improved either metric for these scenarios. Additionally, using l=0.2 for the squared exponential appears to have been reasonable. l=0.5 performed well for some scenarios but was inconsistent and performed poorly for scenarios 3,4 and 7. As expected, the optimal value for l therefore depends on the true underlying

dose-response curve. That said, the CoBe DOA using the squared exponential kernel was often effective in each scenario with multiple different length hyperparameters. This may suggest that effective optimisation using the CoBe DOA does not require precision in the choice of length hyperparameter.

S7.2. CoBe DOA: CCBP Kernel Length Hyperparameters for Prime/Boost and Prime/Boost/Second-Boost administration

In this work the length parameters l=0.2 was used for modelling single-administration dose-response, l_1=l_2=0.25 were used for modelling prime/boost dose-response, and length parameters l_1=l_2=l_3=0.4 were used for modelling prime/boost/second-boost dose-response. We discussed the choice of l=0.2 above. Here we discuss why the values of the length hyperparameters for prime/boost and modelling prime/boost/second-boost were chosen. We note that these were not chosen based on systematic optimization (to avoid advantaging the CoBe DOA relative to the other DOAs), but rather on geometrical principles.

Consider a 1-D dosing domain normalised such that the maximum dose is 1 and the minimum is 0. If a point is selected in the centre (0.5), then a 1-d sphere with radius = l = 0.2 around that point would have 40% of the dosing domain inside.

Consider a 2-D dosing domain normalised such that the maximum dose is 1 and the minimum is 0 for both dimensions. Let l_1=l_2. If the central point is selected in the centre ([0.5, 0.5]), then a 2-d sphere with radius l1 around that point would have $100\pi l_1^2\%$ of the dosing domain inside. For l_1=0.2, this would be 12.5%, so less of the dosing domain would be influenced per data point. Hence, we believed that it would be likely that we should choose l_1=l_2>0.2.

The choice of l_1 such that 40% is inside is approximately .36. Hence choosing l_1=l_2=0.36 would approximately correspond to the same percentage of the dosing domain being in some sense ‘strongly’ influenced by a given data point in the centre of the dosing domain. However, using such a large value of l1 might limit optimisation of the prime or boost dose individually. We therefore choose to use some value between 0.2 and 0.36, so we choose 0.25.

A similar argument was used to choose $l_1=l_2=l_3=0.4$. Consider a 3-D dosing domain normalised such that the maximum dose is 1 and the minimum is 0 for both dimensions. If the central point is selected in the centre ([0.5, 0.5, 0.5]), then a 3-D sphere with radius l_1 around that point would have $100\frac{4\pi}{3}l_1^3\%$ of the dosing domain inside. Hence the choice of l_1 such that 40% is inside would be 0.66. Again, we choose to choose a value between 0.2 and 0.66 as a compromise between optimising within the 3-D dosing domain and being able to optimise along each dimension individually.

We investigated of the effect that this may have on selection of maximum efficacy dose and benefit to trial participants. This was done by following the same methodology as objective 2 of the main body of this work, however we only investigated scenarios 1 and 6. Scenario 1 was used to investigate the choice of $l_1=l_2=0.25$ and scenario 6 was used to investigate the choice of $l_1=l_2=l_3=0.4$. All DOAs investigated were CoBe DOAs but with varying length hyperparameters. We show these results in Figure S3.

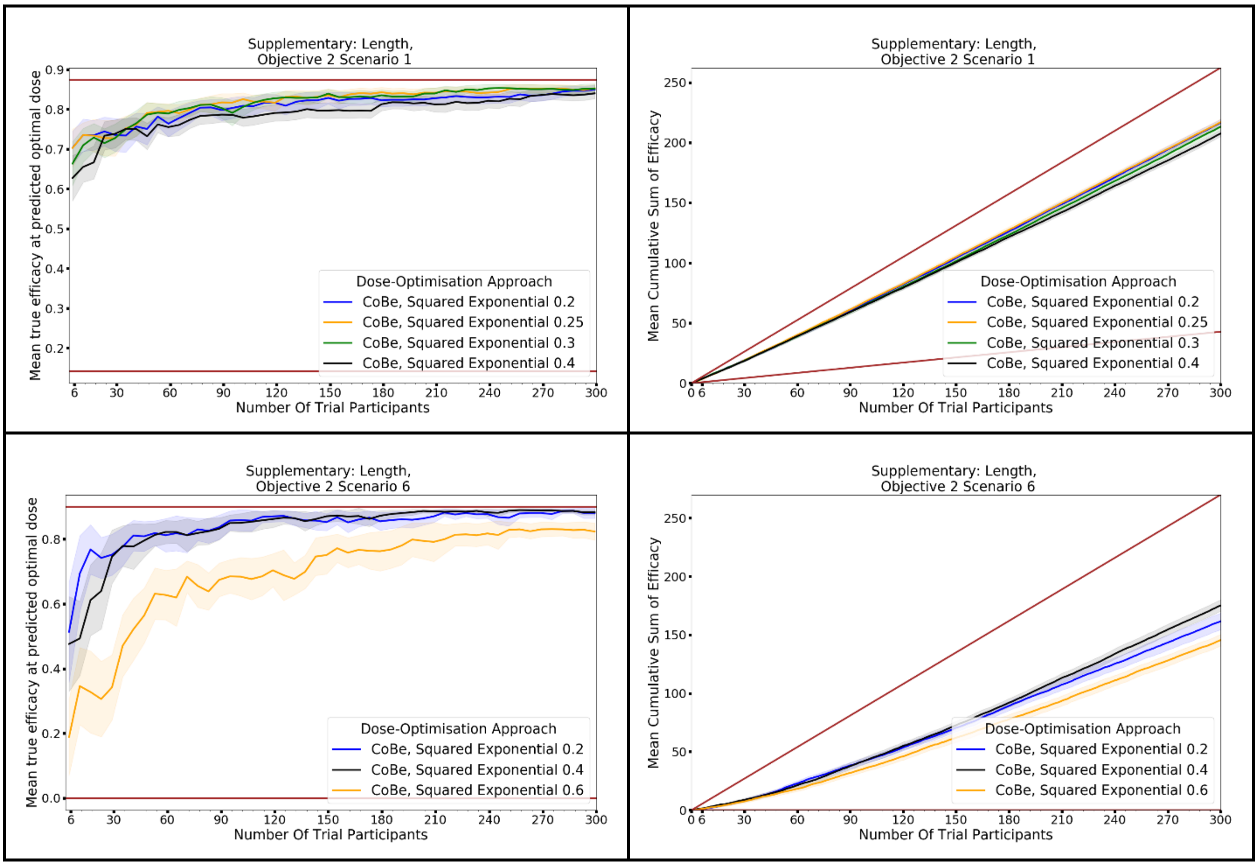

Figure S7.2. Mean true efficacy at the predicted optimal dose (left) and mean cumulative sum of efficacy (right) against trial size for all seven objective S7.2 scenarios (top to bottom). These are the mean values and 95%CI values across 100 simulations. For the true efficacy plots (left), the brown lines show the minimum and maximum possible efficacy that could be achieved in that scenario. For the cumulative efficacy plots (right) the brown lines represent the maximum and minimum cumulative efficacy sum that could be expected for that scenario.

These results suggest our choice of length hyperparameters were reasonable, though our initial concerns based on our geometric interpretation that $l_1=l_2=0.2$ or $l_1=l_2=l_3=0.2$ would be too small may not have been correct. $l_1=l_2=0.2$ and $l_1=l_2=l_3=0.2$ both performed well in this investigation, though this may not have been the case for other scenarios. It also suggests that the performance of the CoBe DOA may not be that sensitive to the choice of length hyperparameter.

S7.3. CoBe DOA: Cohort Size

For the CoBe DOA, and indeed all adaptive trial designs, clinicians must choose how to divide their total trial population into trial cohorts. In this work the N total trial participants were divided into C cohorts of cohort size b, with b = N/C. In the main body of the text the CoBe DOA used N = 300, C = 50, b = 6. There is clearly a trade-off to be made in terms of the size of b. As b decreases, C increases, meaning that a

larger number of cohorts would be required before the trial can be concluded. As b increases however, the DOA becomes less adaptive, with b = N (C=1) being not adaptive at all. Decreasing b increases the time taken to conduct a trial, increasing b may reduce the benefits of adaptive design that we have discussed in this work (increased benefit to trial participants and improved capacity to select optimal dose).

We investigated of the effect that this may have on selection of maximum efficacy dose and benefit to trial participants. This was done by following the same methodology and scenarios as objective 1 of the main body of this work, but where all DOAs investigated were CoBe DOAs with six different values for b. Results are shown in figure S4

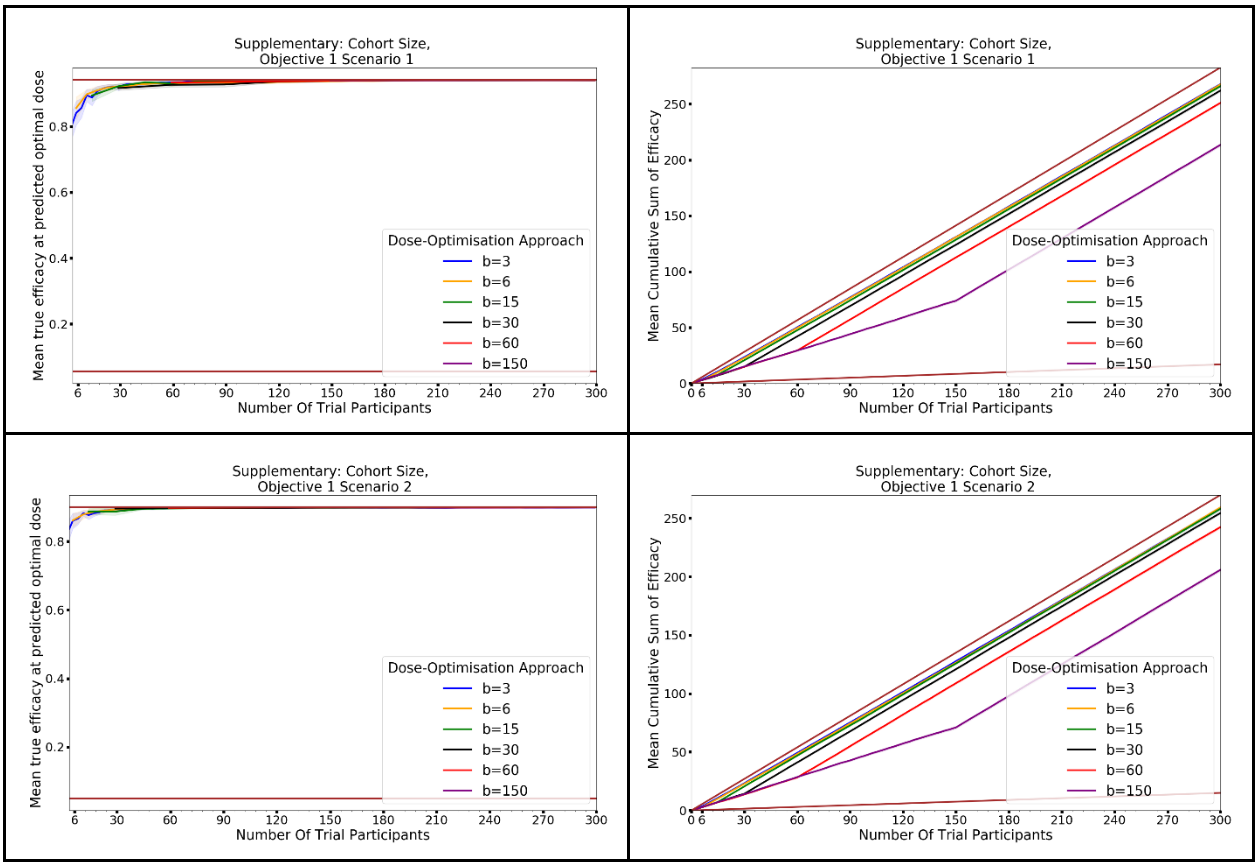

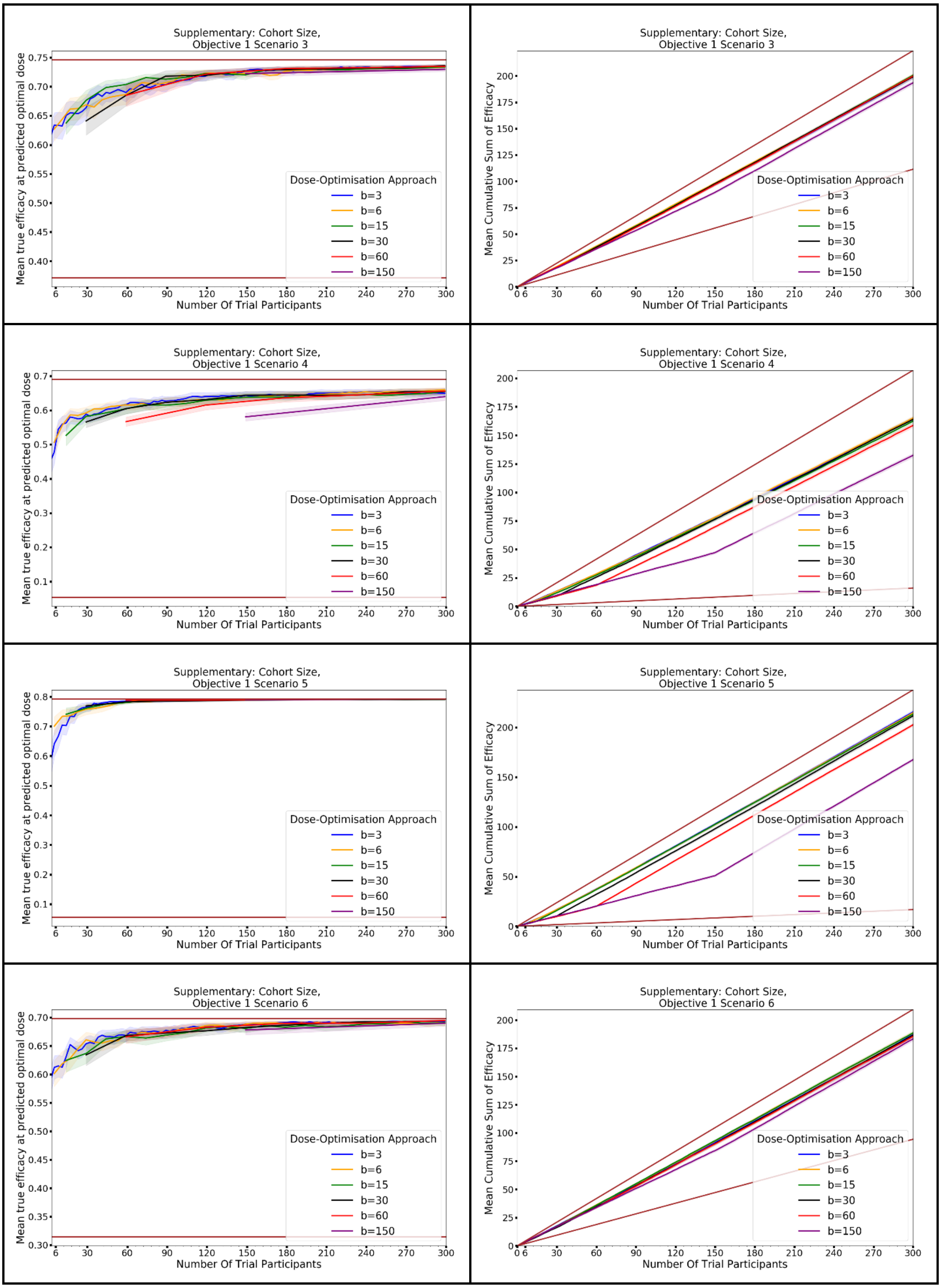

Supplementary: Cohort Size, Objective 1 Scenario 3
Mean true efficacy at predicted optimal dose
Number Of Trial Participants
Dose-Optimisation Approach
b=3
b=6
b=15
b=30
b=60
b=150
Supplementary: Cohort Size, Objective 1 Scenario 3
Mean Cumulative Sum of Efficacy
Number Of Trial Participants
Dose-Optimisation Approach
b=3
b=6
b=15
b=30
b=60
b=150
Supplementary: Cohort Size, Objective 1 Scenario 4
Mean true efficacy at predicted optimal dose
Number Of Trial Participants
Dose-Optimisation Approach
b=3
b=6
b=15
b=30
b=60
b=150
Supplementary: Cohort Size, Objective 1 Scenario 4
Mean Cumulative Sum of Efficacy
Number Of Trial Participants
Dose-Optimisation Approach
b=3
b=6
b=15
b=30
b=60
b=150
Supplementary: Cohort Size, Objective 1 Scenario 5
Mean true efficacy at predicted optimal dose
Number Of Trial Participants
Dose-Optimisation Approach
b=3
b=6
b=15
b=30
b=60
b=150
Supplementary: Cohort Size, Objective 1 Scenario 5
Mean Cumulative Sum of Efficacy
Number Of Trial Participants
Dose-Optimisation Approach
b=3
b=6
b=15
b=30
b=60
b=150
Supplementary: Cohort Size, Objective 1 Scenario 6
Mean true efficacy at predicted optimal dose
Number Of Trial Participants
Dose-Optimisation Approach
b=3
b=6
b=15
b=30
b=60
b=150
Supplementary: Cohort Size, Objective 1 Scenario 6
Mean Cumulative Sum of Efficacy
Number Of Trial Participants
Dose-Optimisation Approach
b=3
b=6
b=15
b=30
b=60
b=150

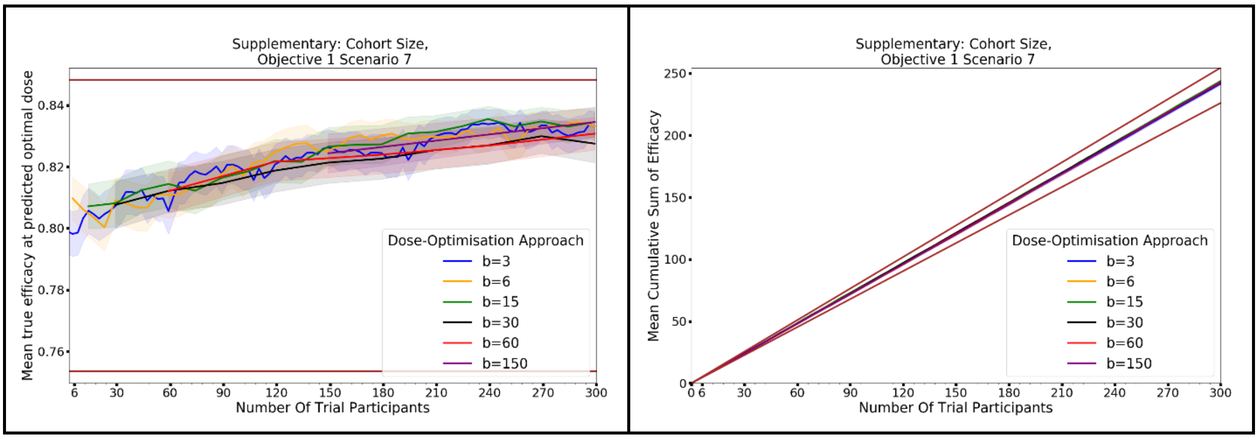

Figure S7.3. Mean true efficacy at the predicted optimal dose (left) and mean cumulative sum of efficacy (right) against trial size for all seven objective S7.3 scenarios (top to bottom). These are the mean values and 95%CI values across 100 simulations. For the true efficacy plots (left), the brown lines show the minimum and maximum possible efficacy that could be achieved in that scenario. For the cumulative efficacy plots (right) the brown lines represent the maximum and minimum cumulative efficacy sum that could be expected for that scenario.

The results of this investigation were as expected. Using fewer, larger cohorts led to a reduction in cumulative sum of efficacy, though the gradient of the cumulative sum of efficacy for all DOAs was typically equal for each of the DOAs after their first complete cohort. For some scenarios we observed that there was also a reduction in mean true utility at the predicted optimal. For example, for scenario 4 after 60 trial participants, the CoBe DOAs with b = 3, 6, 15, and 30 all outperformed the CoBe DOA that had conducted 1 cohort of b = 60. From a qualitative inspection, the CoBe should use at least three cohorts (C>2, b<N/2) to maximise vaccine efficacy, and that a large number of cohorts should be used to maximise benefit to trial participants. This may however vary by scenario. The trade-off between this and reducing the time requirements of vaccine clinical trials would require consideration with vaccine developers, but we hope that this section has shown that our results were unlikely to be biased by our choice of b=6.

S7.4. Parametric DOA: SoftMax temperature hyperparameter

The Parametric DOA used a SoftMax selection function as the method of trial selection, which we have previously shown to be effective for addressing potential concerns relating to the exploration/exploitation trade-off [3]. As discussed, this selection method depends on an 'inverse-temperature' hyperparameter t, which in the main body of this work we set as inverse_temperature t = 6.9. Increasing the value of inverse-temperature would make the parametric DOA more exploitative.

Decreasing the value of inverse-temperature would make the parametric DOA more explorative.

We investigated the effect that this may have on selection of maximum efficacy dose and benefit to trial participants. This was done by following the same methodology and scenarios as objective 1 of the main body of this work, but where all DOAs investigated were parametric DOAs with five different inverse-temperature hyper parameters. Results are shown in Figure S5.

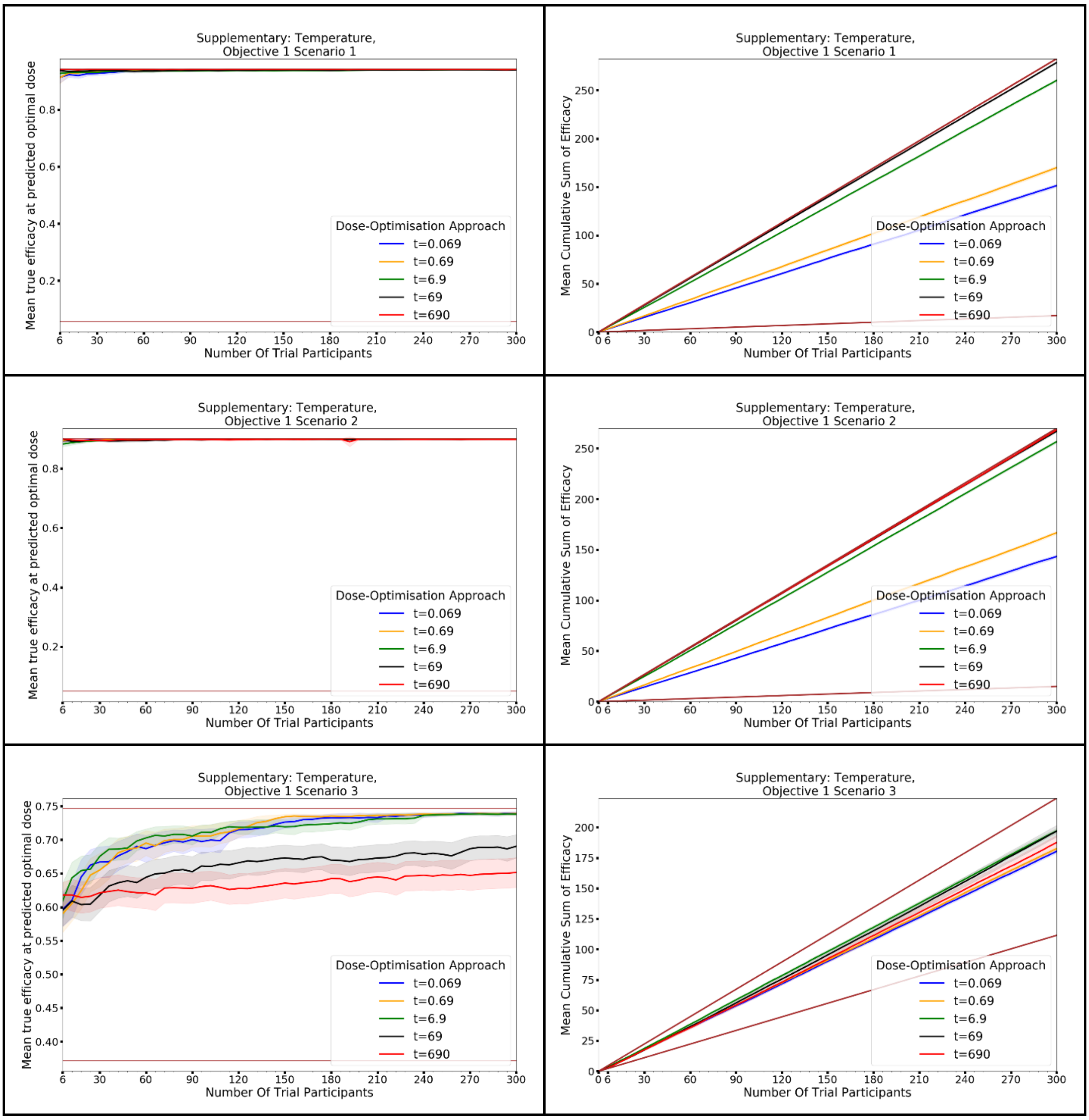

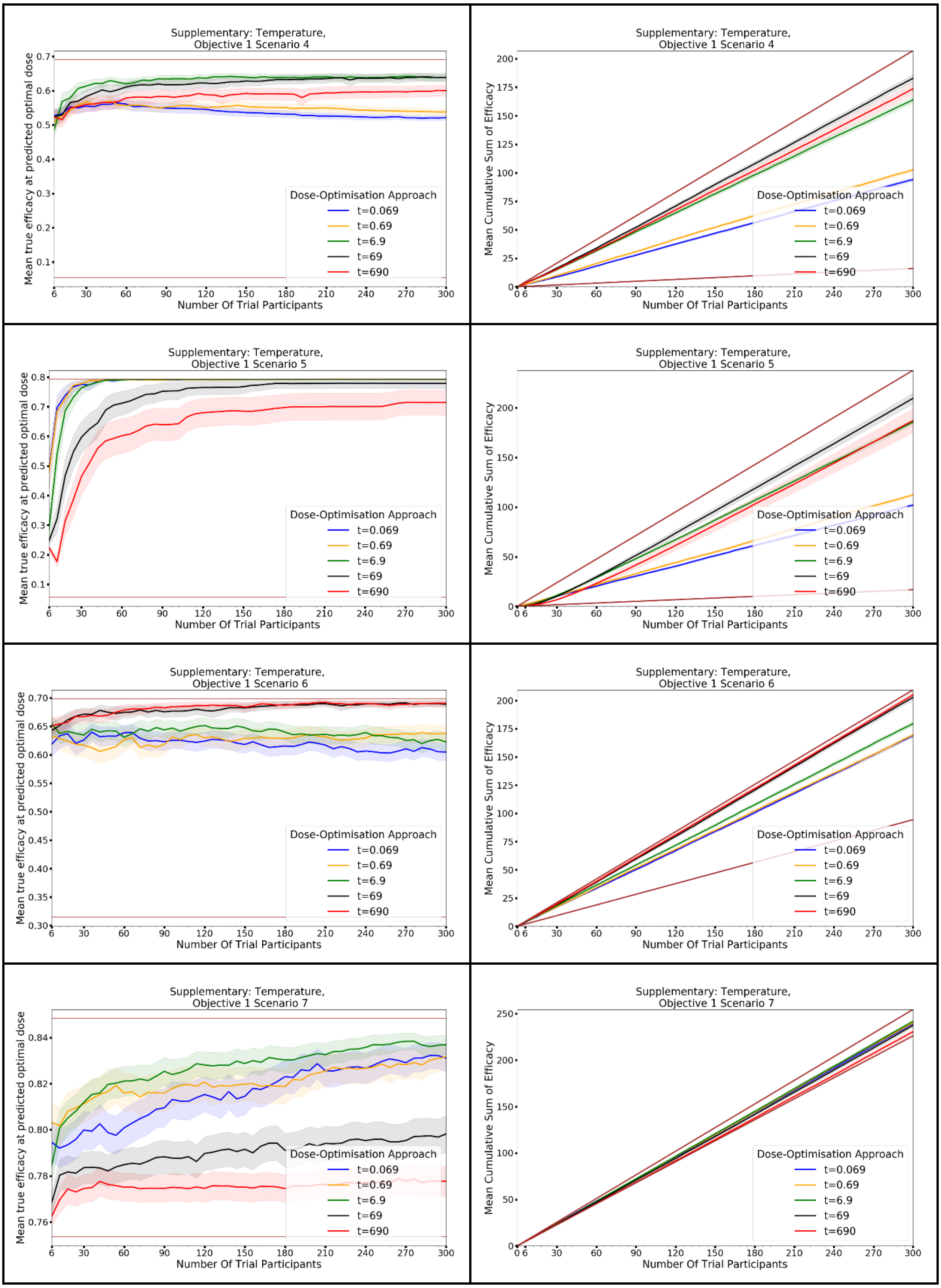

Figure S7.4. Mean true efficacy at the predicted optimal dose (left) and mean cumulative sum of efficacy (right) against trial size for all seven objective S7.4 scenarios (top to bottom). These are the mean values and 95%CI values across 100 simulations. For the true efficacy plots (left), the

brown lines show the minimum and maximum possible efficacy that could be achieved in that scenario. For the cumulative efficacy plots (right) the brown lines represent the maximum and minimum cumulative efficacy sum that could be expected for that scenario.

As expected, for different scenarios the ‘optimal’ value for inverse temperature varied. For example, t = 690 was best able to locate a maximally efficacious dose for scenario 6, but was worst for scenario 7. For t = 0.069 and t = 0.69, the mean cumulative sum of efficacy was typically far lower for all numbers of trial participants compared to when larger values of t were used. We believe that the results for t=6.9 show that it was typically optimal or near optimal for all scenarios other than scenario 6. Given this finding, we believe that this choice of t was reasonable in the main body of work, and that the effectiveness of the parametric DOA was not underestimated due to our choice of t=6.9. This supplementary section agrees with previous finding that parametric modelling-based adaptive design should not typically use a highly exploitative method of trial dose selection.

S7.5. Uniform Naive DOA: Number of Doses

For the uniform naive DOA, we had the number of dosing groups that would be investigated by the DOA, b. We choose b = 6 for single dose administration, b = 9 for prime/boost administration and b=27 for prime/boost/second-boost administration. Investigating only a small number of dosing groups could limit vaccine efficacy/utility, as if none of the b doses are optimal then the true optimal dose could not be selected. On the other hand, if b is too large then there is a reduction in the number of individuals available per dosing group. For example, if 30 dosing groups were investigated with a total number of trial participants N = 120, then only 4 individuals would be tested per group, which may not be sufficient to determine which dose is optimal.

We investigated of the effect that changing b might have on selection of maximum efficacy dose and benefit to trial participants. This was done by following the same methodology and scenarios as objective 1 of the main body of this work, but where all DOAs investigated were Uniform Naive DOAs with six different values for b. The doses investigated for each value of b are given in table S1, with doses being as evenly distributed across the dosing domain as possible. Results are shown in figure S6.

Number of dosing groups (b)	Doses investigated
1	0.5
2	0.33, 0.66
3	0.0, 0.5, 1.0
4	0.0, 0.33, 0.66, 1.0
5	0.0, 0.25, 0.5, 0.75, 1.0
6	0.0, 0.2, 0.4, 0.6, 0.8, 1.0
10	0.0, 0.11, 0.22, 0.33, 0.44, 0.55, 0.66, 0.77, 0.88, 1.0

Table S7.5. Doses investigated depending on b.

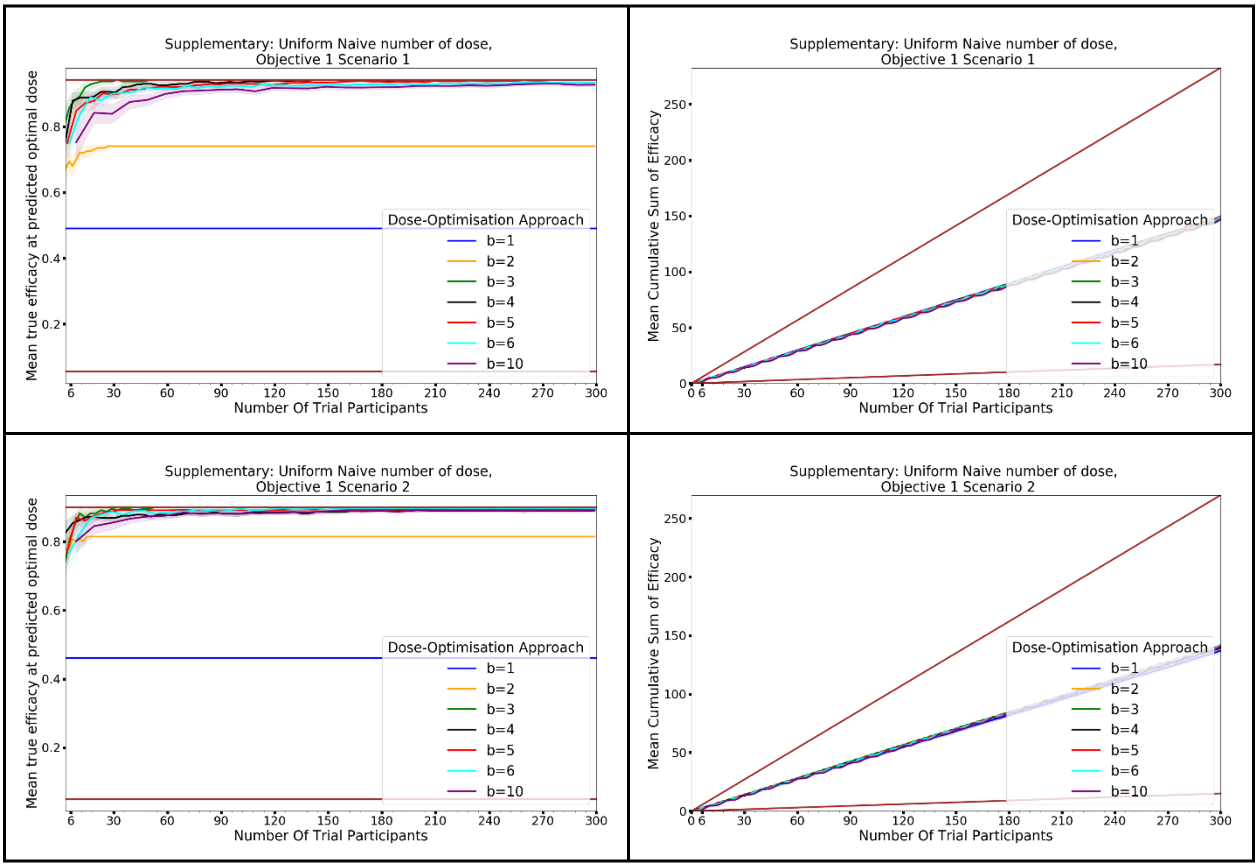

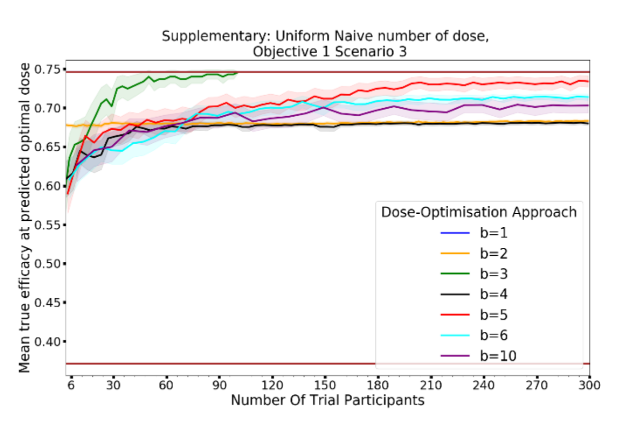
Supplementary: Uniform Naive number of dose,
Objective 1 Scenario 3
Mean true efficacy at predicted optimal dose
Number Of Trial Participants
Dose-Optimisation Approach
b=1
b=2
b=3
b=4
b=5
b=6
b=10

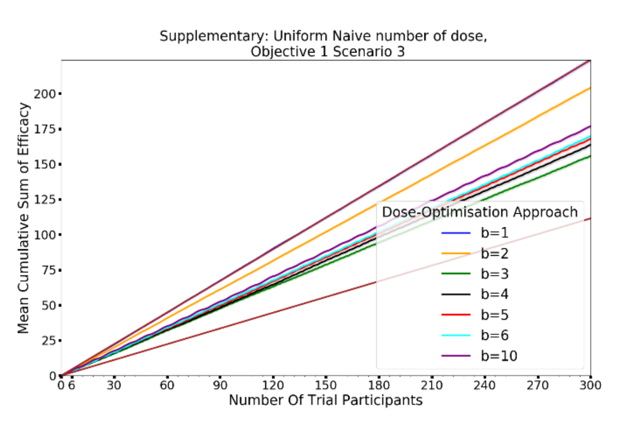
Supplementary: Uniform Naive number of dose,
Objective 1 Scenario 3
Mean Cumulative Sum of Efficacy
Number Of Trial Participants
Dose-Optimisation Approach
b=1
b=2
b=3
b=4
b=5
b=6
b=10

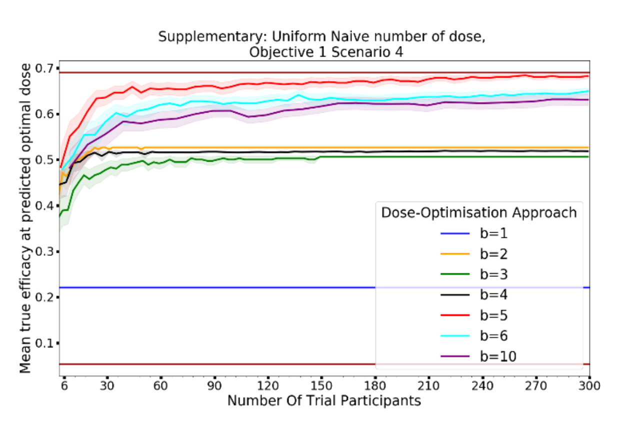
Supplementary: Uniform Naive number of dose,
Objective 1 Scenario 4
Mean true efficacy at predicted optimal dose
Number Of Trial Participants
Dose-Optimisation Approach
b=1
b=2
b=3
b=4
b=5
b=6
b=10

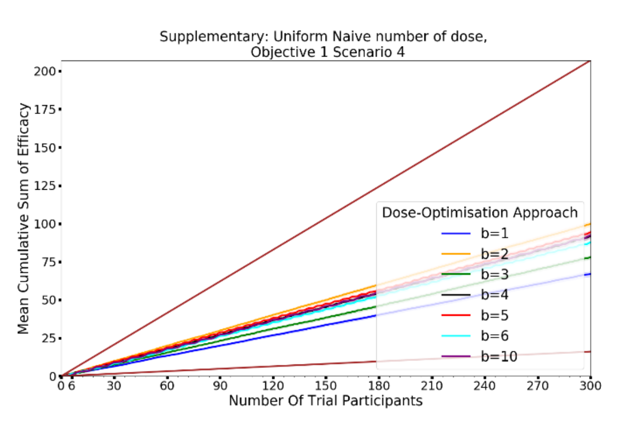
Supplementary: Uniform Naive number of dose,
Objective 1 Scenario 4
Mean Cumulative Sum of Efficacy
Number Of Trial Participants
Dose-Optimisation Approach
b=1
b=2
b=3
b=4
b=5
b=6
b=10

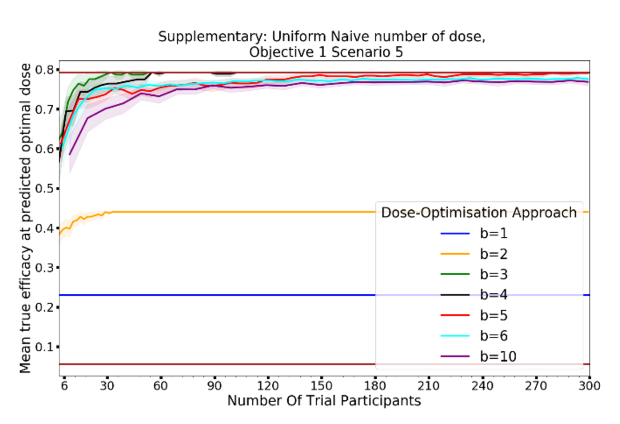
Supplementary: Uniform Naive number of dose,
Objective 1 Scenario 5
Mean true efficacy at predicted optimal dose
Number Of Trial Participants
Dose-Optimisation Approach
b=1
b=2
b=3
b=4
b=5
b=6
b=10

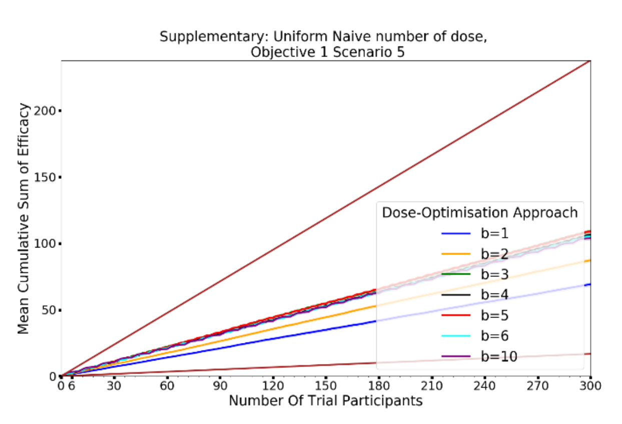
Supplementary: Uniform Naive number of dose,
Objective 1 Scenario 5
Mean Cumulative Sum of Efficacy
Number Of Trial Participants
Dose-Optimisation Approach
b=1
b=2
b=3
b=4
b=5
b=6
b=10

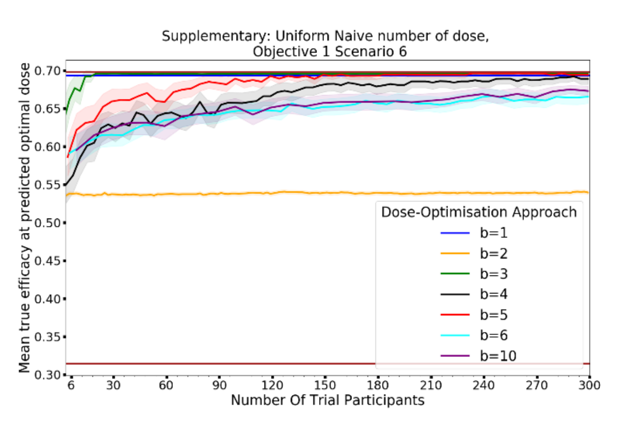
Supplementary: Uniform Naive number of dose,
Objective 1 Scenario 6
Mean true efficacy at predicted optimal dose
Number Of Trial Participants
Dose-Optimisation Approach
b=1
b=2
b=3
b=4
b=5
b=6
b=10

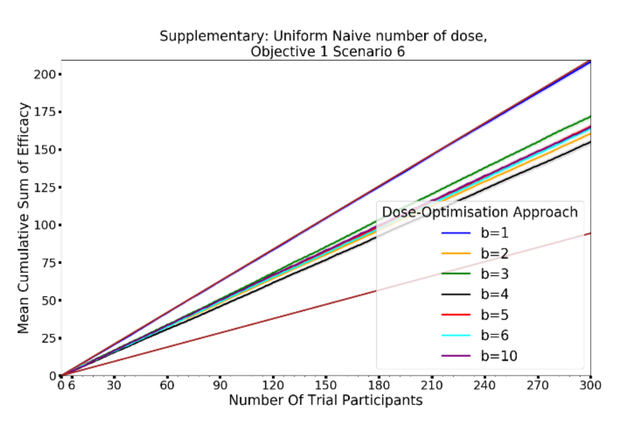
Supplementary: Uniform Naive number of dose,
Objective 1 Scenario 6
Mean Cumulative Sum of Efficacy
Number Of Trial Participants
Dose-Optimisation Approach
b=1
b=2
b=3
b=4
b=5
b=6
b=10

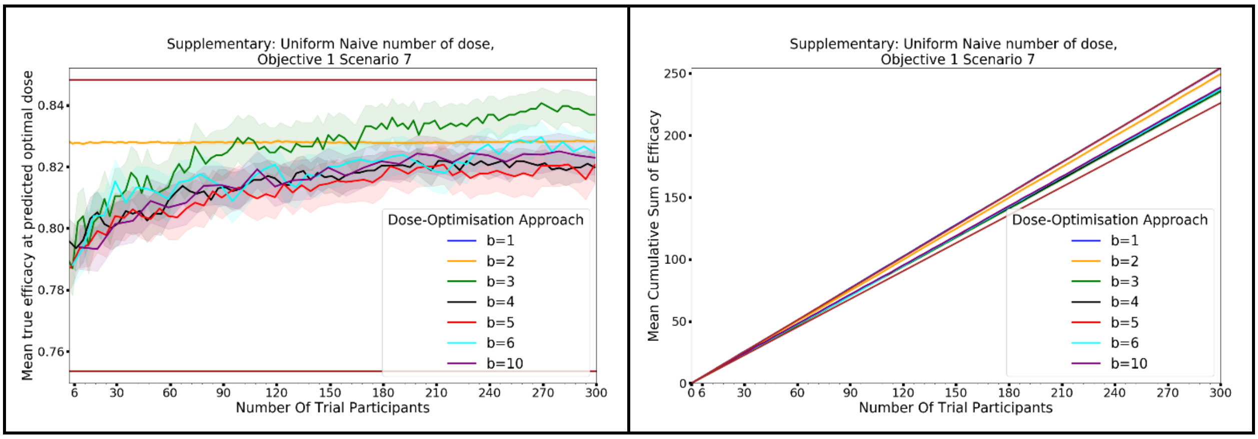

Figure S7.5. Mean true efficacy at the predicted optimal dose (left) and mean cumulative sum of efficacy (right) against trial size for all seven objective S7.5 scenarios (top to bottom). These are the mean values and 95%CI values across 100 simulations. For the true efficacy plots (left), the brown lines show the minimum and maximum possible efficacy that could be achieved in that scenario. For the cumulative efficacy plots (right) the brown lines represent the maximum and minimum cumulative efficacy sum that could be expected for that scenario.

The results of this investigation were clearly biased in a way that could not easily be controlled for. How well the Uniform Naive DOA performed for locating maximum efficacy dose depended on the scenario. For example, the Uniform Naive DOA with b=1 'locates' the optimal dose immediately for scenario 3, as the only dose investigated is 0.5 and this is indeed the true optimal dose. b = 3 also locates the optimal dose very well for this scenario, whereas b = 2 performs very poorly and neither 0.33 nor 0.66 are near the true optimal dose. This is not a reflection that using two dosing groups is in general worse than using one dosing group or three dosing groups, but a reflection that the performance of the uniform naive DOA is dependent on how optimal the doses it investigates are. This sensitivity is particularly true for small b. We choose b=6 as that is the number of dosing groups that was used in [6], and because investigation of at least 5 or 6 dosing groups was suggested by [7].

There was trivial difference in cumulative sum of efficacy, as none of these DOAs used adaptive design.

S7.6. Adaptive Naive DOA: Number of Doses

This section is exactly the same as discussed for the Uniform Naive DOA, except using the Adaptive Naive DOA. Results are shown in figure S7.

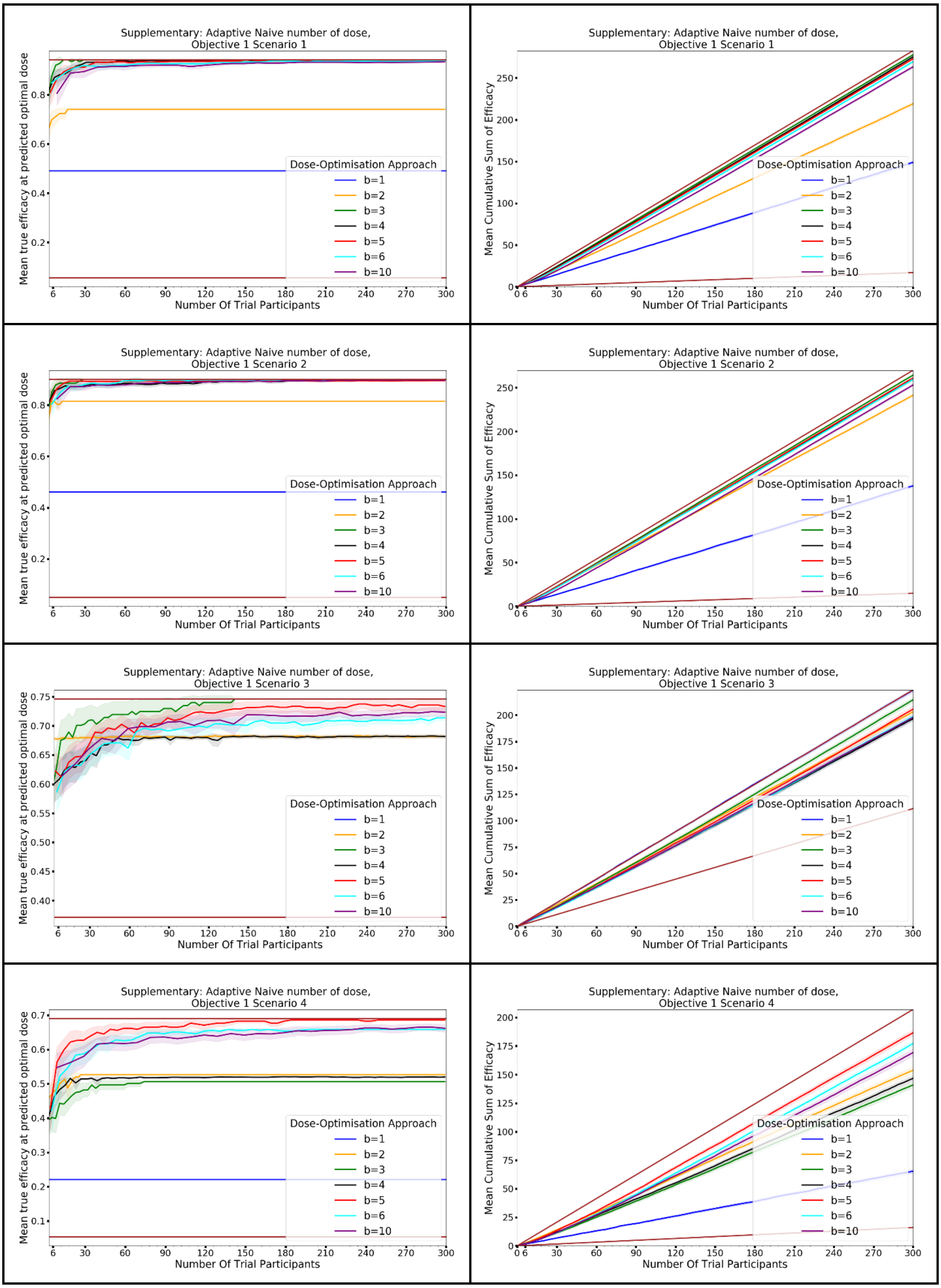
Supplementary: Adaptive Naive number of dose,
Objective 1 Scenario 1
Mean true efficacy at predicted optimal dose
Number Of Trial Participants
Dose-Optimisation Approach
b=1
b=2
b=3
b=4
b=5
b=6
b=10
Supplementary: Adaptive Naive number of dose,
Objective 1 Scenario 1
Mean Cumulative Sum of Efficacy
Number Of Trial Participants
Dose-Optimisation Approach
b=1
b=2
b=3
b=4
b=5
b=6
b=10
Supplementary: Adaptive Naive number of dose,
Objective 1 Scenario 2
Mean true efficacy at predicted optimal dose
Number Of Trial Participants
Dose-Optimisation Approach
b=1
b=2
b=3
b=4
b=5
b=6
b=10
Supplementary: Adaptive Naive number of dose,
Objective 1 Scenario 2
Mean Cumulative Sum of Efficacy
Number Of Trial Participants
Dose-Optimisation Approach
b=1
b=2
b=3
b=4
b=5
b=6
b=10
Supplementary: Adaptive Naive number of dose,
Objective 1 Scenario 3
Mean true efficacy at predicted optimal dose
Number Of Trial Participants
Dose-Optimisation Approach
b=1
b=2
b=3
b=4
b=5
b=6
b=10
Supplementary: Adaptive Naive number of dose,
Objective 1 Scenario 3
Mean Cumulative Sum of Efficacy
Number Of Trial Participants
Dose-Optimisation Approach
b=1
b=2
b=3
b=4
b=5
b=6
b=10
Supplementary: Adaptive Naive number of dose,
Objective 1 Scenario 4
Mean true efficacy at predicted optimal dose
Number Of Trial Participants
Dose-Optimisation Approach
b=1
b=2
b=3
b=4
b=5
b=6
b=10
Supplementary: Adaptive Naive number of dose,
Objective 1 Scenario 4
Mean Cumulative Sum of Efficacy
Number Of Trial Participants
Dose-Optimisation Approach
b=1
b=2
b=3
b=4
b=5
b=6
b=10

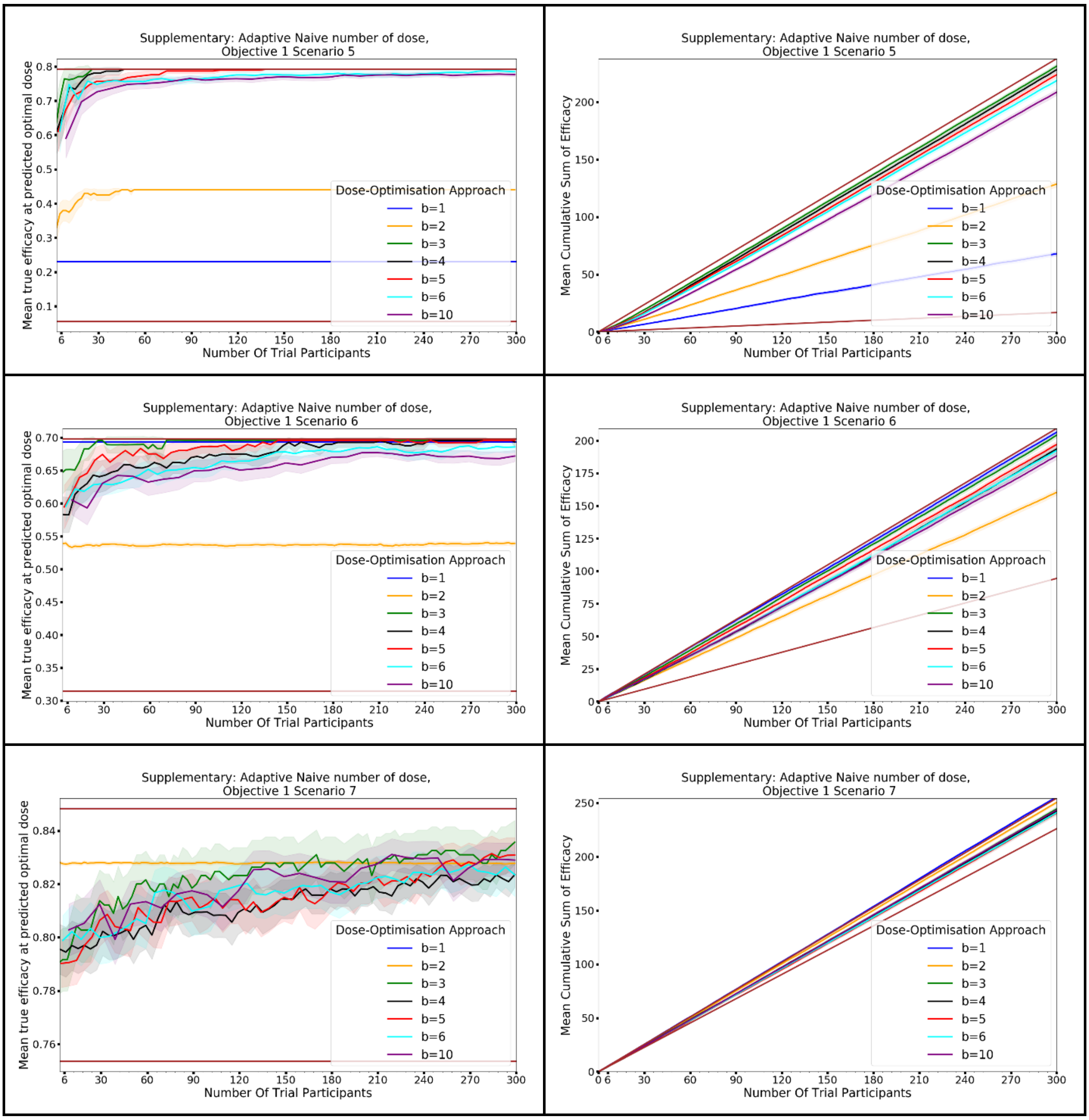

Figure S7.6. Mean true efficacy at the predicted optimal dose (left) and mean cumulative sum of efficacy (right) against trial size for all seven objective S7.6 scenarios (top to bottom). These are the mean values and 95%CI values across 100 simulations. For the true efficacy plots (left), the brown lines show the minimum and maximum possible efficacy that could be achieved in that scenario. For the cumulative efficacy plots (right) the brown lines represent the maximum and minimum cumulative efficacy sum that could be expected for that scenario.

Findings were similar to those for the Uniform Naive DOA, with the performance of each DOA being dependent largely on whether any of the doses that were investigated were indeed optimal. There was a difference between the DOAs with regards to cumulative sum of efficacy, however again this was dependent on the scenario. For example, b=1 and b=3 had large cumulative sums of efficacy for scenario 3, with the

Uniform Naive DOA that had b=1 giving the optimal dose of 0.5 to all individuals. We would argue that this does not reflect consistent capacity for dose optimisation and ethical trial design, especially given the inferior performance of this DOA for all environments where the one investigated dose was not optimal. b=6 was more consistent across different scenarios, justifying our choice.

Paper 5 Supplementary References

1. Thall, P.F.; Cook, J.D. Dose-Finding Based on Efficacy-Toxicity Trade-Offs. *Biometrics* **2004**, *60*, 684–693, doi:10.1111/j.0006-341X.2004.00218.x.
2. Brock, K.; Billingham, L.; Copland, M.; Siddique, S.; Sirovica, M.; Yap, C. Implementing the EffTox Dose-Finding Design in the Matchpoint Trial. *BMC Medical Research Methodology* **2017**, *17*, 112, doi:10.1186/s12874-017-0381-x.
3. Benest, J.; Rhodes, S.; Evans, T.G.; White, R.G. Mathematical Modelling for Optimal Vaccine Dose Finding: Maximising Efficacy and Minimising Toxicity. *Vaccines (Basel)* **2022**, *10*, 756, doi:10.3390/vaccines10050756.
4. Thall, P.F. BAYESIAN ADAPTIVE DOSE-FINDING BASED ON EFFICACY AND TOXICITY. *Journal of Statistical Research* **2012**, *46*, 187–202.
5. Rolland, P.; Kavis, A.; Immer, A.; Singla, A.; Cevher, V. Efficient Learning of Smooth Probability Functions from Bernoulli Tests with Guarantees. In Proceedings of the Proceedings of the 36th International Conference on Machine Learning; PMLR, May 24 2019; pp. 5459–5467.
6. Takahashi, A.; Suzuki, T. Bayesian Optimization Design for Dose-Finding Based on Toxicity and Efficacy Outcomes in Phase I/II Clinical Trials. *Pharmaceutical Statistics* **2021**, *20*, 422–439, doi:10.1002/pst.2085.
7. Diniz, M.A.; Tighiouart, M.; Rogatko, A. Comparison between Continuous and Discrete Doses for Model Based Designs in Cancer Dose Finding. *PLOS ONE* **2019**, *14*, e0210139, doi:10.1371/journal.pone.0210139.
